# Long-Range Charge Transport via Redox Ligands in Quantum Dot Assemblies

**DOI:** 10.1021/acsnano.2c09192

**Published:** 2022-12-14

**Authors:** Yan B. Vogel, Maarten Stam, Jence T. Mulder, Arjan J. Houtepen

**Affiliations:** Optoelectronic Materials Section, Faculty of Applied Sciences, Delft University of Technology, Van der Maasweg 9, 2629 HZDelft, The Netherlands

**Keywords:** quantum dots, charge transport, charge transfer, redox ligands, electrochemistry

## Abstract

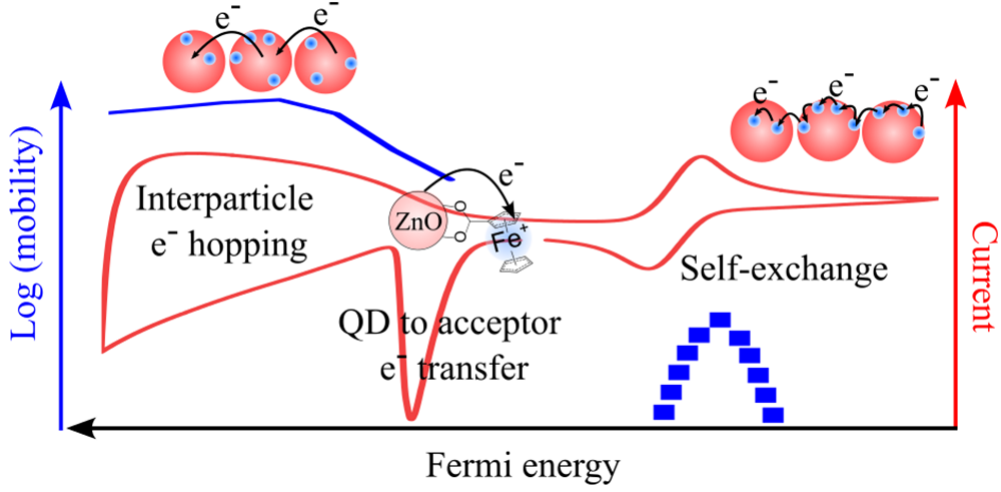

We present a strategy to actively engineer long-range charge transport in colloidal quantum dot assemblies by using ligand functionalities that introduce electronic states and provide a path for carrier transfer. This is a shift away from the use of inactive spacers to modulate charge transport through the lowering of the tunneling barrier for interparticle carrier transfer. This is accomplished with the use of electronically coupled redox ligands by which a self-exchange chain reaction takes place and long-range charge transport is enabled across the film. We identified the different modes of charge transport in these quantum dot/redox ligand assemblies, their energetic position and kinetics, and explain how to rationally manipulate them through modulation of the Fermi level and redox ligand coverage.

## Introduction

Quantum dots (QDs) show great promise for next-generation semiconductor applications^1^ such as solar cells,^2^ photocatalysts,^3^ and sensors.^4^ For such devices, QDs are required to be assembled in films, in which charge transport across the assembly determines their performance. For this reason, over the past two decades a wealth of studies have been conducted to gain a better understanding of the mechanisms of charge transport in QD assemblies and how to control them.^1,5−9^ Most commonly, long aliphatic ligands are replaced with shorter ones, which decreases the interparticle distance and increases the electron hopping rate exponentially,^6,10,11^ while certain inorganic ligands^12^ and conjugated molecules also lower the tunneling barrier.^13^

In all the above cases, the introduced ligands act as passive spacers. In contrast, the introduction of electronic states could be used as active units to open up an additional path of charge transport, but this possibility remained until now unexplored. Here, we engineer electronic states on the QD surface through the anchoring of redox ligands with well-defined states that provide sites for charge transport. We use an electrochemical approach that allows to simultaneously access the narrow redox states by fine modulation of the Fermi level and measure the kinetics of charge transport.

We show that charge transport across these QD/redox ligand assemblies takes place via two complementary pathways: electron hopping through the conduction band of the QDs and by self-exchange through the immobile redox ligands. Long-range charge transport is accompanied by charge transfer between QDs and redox ligands, and ion transport. The rate of each of the individual events can be controlled and manipulated in a systematic way and have different dependences on the studied variables. For example, while both ion transport and electron hopping are independent of the redox ligand concentration, self-exchange is strongly affected as predicted by percolation theory. In all the investigated scenarios, ion transport is faster than self-exchange, which makes this experimental model ideal to test theories of charge transport through self-exchange in QD/redox ligand assemblies.

## Results and Discussion

### Development of the Experimental Model

**Figure 1:**
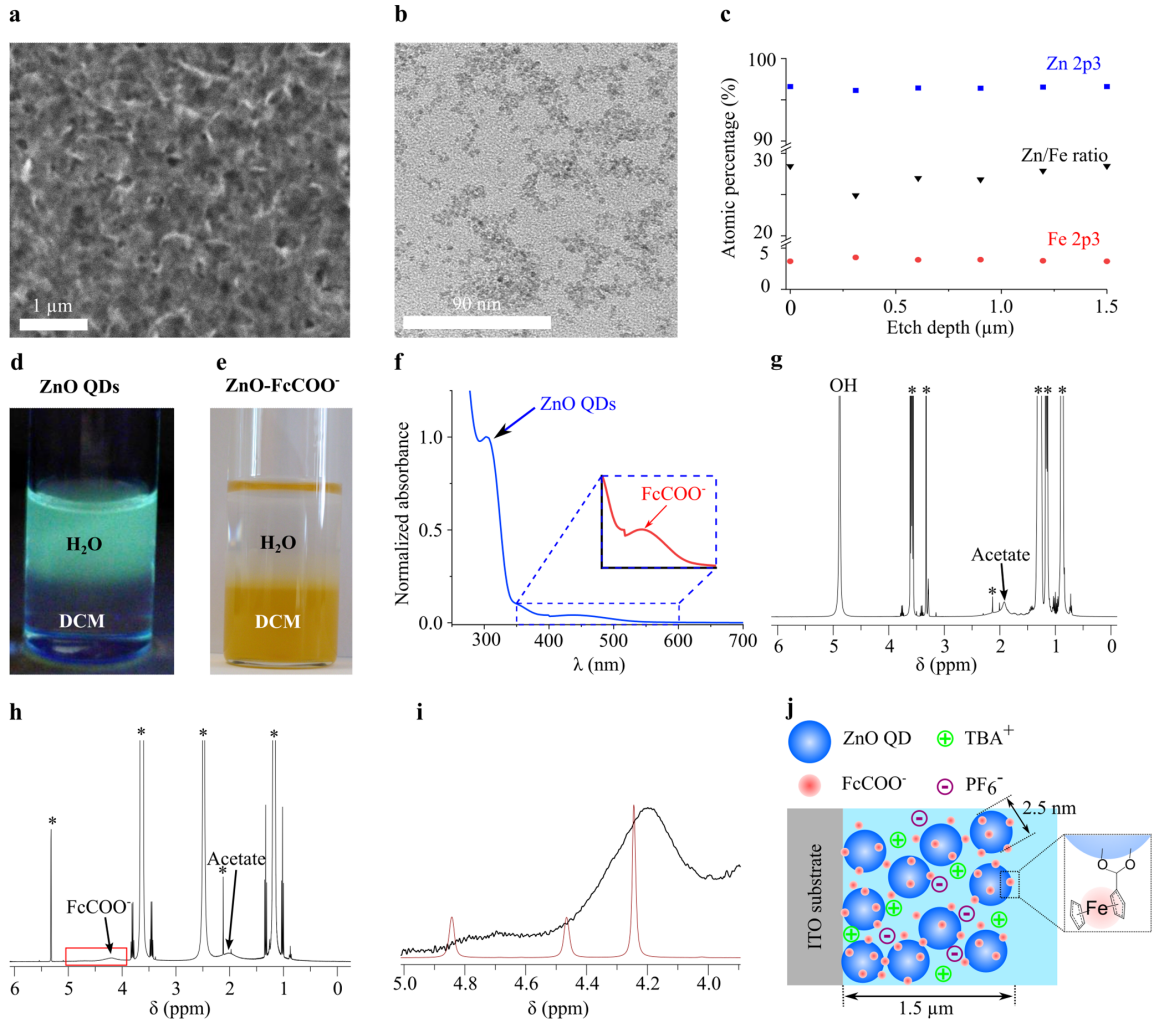
Characterization of the experimental model. (a) SEM image of a ZnO-FcCOO^–^ film. (b) TEM image of the ZnO-FcCOO^–^ QDs. (c) XPS depth-profile of a ZnO-FcCOO^–^ film indicating the atomic percentage of Zn and Fe, and Zn/Fe ratio, as a function of the etch depth. The atomic percentages were obtained from the Zn 2p3 and Fe 2p3 XPS peaks (see Figure S3). (d,e) Dichloromethane (DCM)/water (H_2_O) biphasic system with (d) ZnO QDs under UV light irradiation and (e) ZnO-FcCOO^–^ QDs. (f) Absorption spectrum of a ZnO-FcCOO^–^ dispersion in dichloromethane. The inset shows the FcCOO^–^ peak. (g–i) NMR spectra of (g) ZnO QDs in CD_3_OD, (h) ZnO-FcCOO^–^ QDs in CD_2_Cl_2_, and (i) ZnO-FcCOO^–^ QDs (black line) and FcCOOH (red line) in CD_2_Cl_2_ in the 4.0–5.0 ppm region. The asterisks in the NMR spectra indicate solvent peaks. (j) Schematic representation of the ZnO-FcCOO^–^ film used in this study to investigate charge transport. The scheme is not to scale. TBA^+^ is the short for tetrabutylammonium cation and PF_6_^–^ is hexafluorophosphate. FcCOOH and FcCOO^–^ are the short forms of ferrocene carboxylic acid and ferrocene carboxylate, respectively.

To test the hypothesis that redox ligands can be used as active entities to engineer charge transport in QD assemblies, we developed an experimental model consisting of ZnO QDs with anchored ferrocene carboxylate (FcCOO^–^) ligands (referred as ZnO-FcCOO^–^ hereafter). FcCOO^–^ was chosen because of the ideal behavior of ferrocene (Fc) as an outer-sphere one-electron redox couple,^14,15^ with the carboxylate group offering an anchoring point to the ZnO,^16^ while ZnO QDs exhibit reversible and stable electrochemical charging/discharging,^17^ allowing for in-depth charge transport studies. The synthesized ZnO QDs have a bandgap of 3.86 eV, as determined by steady-state spectroscopy of a dispersion in ethanol (Figure S1). The ZnO QDs were drop-cast onto indium–tin-oxide (ITO) on glass substrates. These films have a thickness of 1.5 μm, as obtained by profilometry (Figure S2), and a porosity of ∼50% (see Methods). The ZnO QD films were functionalized with FcCOO^–^ moieties by immersion into a solution of ferrocene carboxylic acid (FcCOOH) in acetonitrile overnight and thorough rinsing with acetonitrile. A scanning electron microscopy (SEM) image of the ZnO-FcCOO^–^ film shows a homogeneous and porous film (Figure 1a). A transmission electron microscopy (TEM) image of a dispersion of ZnO-FcCOO^–^, prepared by mixing ethanolic solutions of ZnO QDs and FcCOOH (*vide infra*), shows the individual ZnO-FcCOO^–^ with a diameter of 2.5 nm (Figure 1b). We performed depth-profile X-ray photoelectron spectroscopy (XPS) to the ZnO-FcCOO^–^ film to determine the distribution of FcCOO^–^ through the film. The XPS shows presence of Fe and Zn (see Figure S3), with a constant atomic percentage of Fe and Zn at different depths in the ZnO-FcCOO^–^ film (Figure 1c). The estimated Zn/Fe ratio is ∼30, which corresponds to ∼10 FcCOO^–^ ligands per QD.

We performed a set of experiments (Figure 1d–i) to confirm successful anchoring of FcCOO^–^ onto the ZnO QDs and absence of free (unbound) FcCOOH. First, mixing two ethanolic solutions of FcCOOH and ZnO QDs leads to the formation of an orange precipitate and a discoloration of the ethanolic solution, pointing to attachment of FcCOO^–^ to the ZnO QDs (see Figure S4). The orange product was cleaned thoroughly with ethanol/dichloromethane to remove any free unreacted FcCOOH and ZnO QDs and redispersed in dichloromethane. A biphasic mixture of dichloromethane/water shows that the ZnO QDs with the native ligands transfer to the aqueous phase (Figure 1d) while the orange precipitate transfers to the dichloromethane phase (Figure 1e). Because the ZnO QDs are soluble in water but insoluble in dichloromethane and Fc is soluble in dichloromethane but insoluble in water, this strongly suggests successful anchoring of FcCOO^–^ to the ZnO QDs. Figure 1f shows an absorption spectrum of the cleaned ZnO-FcCOO^–^ dispersed in dichloromethane: the peak at 440 nm corresponds to FcCOO^–^ (Figure S5), while the peak at 325 nm corresponds to ZnO (Figure S1), indicating the presence of both ZnO and FcCOO^–^ in the product. Electrochemical control experiments of solutions in which ZnO-FcCOO^–^ films were immersed overnight do not show any signal due to FcCOO^–^ and therefore demonstrate the absence of free FcCOO^–^ in solution (Figure S6), while electrochemical experiments of the ZnO-FcCOO^–^ films do show presence of FcCOO^–^ (see next section).

Nuclear magnetic resonance (NMR) spectroscopy is a commonly used technique to identify ligand binding to nanoparticles, reflected by a broadening of the NMR peaks due to the slower molecular tumbling of the anchored ligands as compared to free molecules in solution.^18^ An NMR spectrum of the ZnO QDs with the native ligands dispersed in CD_3_OD (Figure 1g) shows the presence of two broad peaks due to acetate (1.92 ppm) and OH^–^ (4.88 ppm) ligands. Some unbound acetate and OH^–^ are also present as evidenced by the sharp singlet at 2.01 ppm and a peak overlaying the broad OH peak at 4.88 ppm, respectively. Acetate and OH^–^ are expected ligands because of the use of zinc acetate and potassium hydroxide during synthesis. All other peaks have been identified as solvent peaks used during workup and have been marked with an asterisk in Figure 1g,h. An NMR spectrum of the orange product in CD_2_Cl_2_ (Figure 1h) shows an additional broad peak in the 4–5 ppm range which coincides with the location of the cyclopentadienyl peaks of FcCOOH (2H 4.84 ppm (s); 2H 4.47 ppm (s); 5H 4.24 ppm (s), Figure 1i). We note the absence of any sharp peak due to unbound FcCOOH in the ZnO-FcCOO^–^ product. We also observe a decrease in the acetate peak and absence of the OH^–^ peak (which should appear at 1.52 ppm in CD_2_Cl_2_) for the ZnO-FcCOO^–^. Taken together, the NMR spectra in Figure 1g–i demonstrate partial ligand exchange of the native ligands with strong binding of FcCOO^–^ to the ZnO QDs and absence of unbound FcCOOH.

Figure 1j is a schematic representation of the developed experimental model based on the NMR spectra demonstrating FcCOO^–^ attachment to the ZnO QDs, the XPS depth-profile showing an even distribution of FcCOO^–^ across the film, and the TEM and SEM images indicating the ZnO-FcCOO^–^ film morphology. This scheme shows the ZnO-FcCOO^–^ film immersed in a solution of acetonitrile (blue background) containing tetrabutylammonium cations (TBA^+^) and hexafluorophosphate anions (PF_6_^–^) that was used throughout this study to investigate the electronic properties of QD/redox ligand assemblies.

### Electronic Properties of QD/Redox Ligand Assemblies

**Figure 2:**
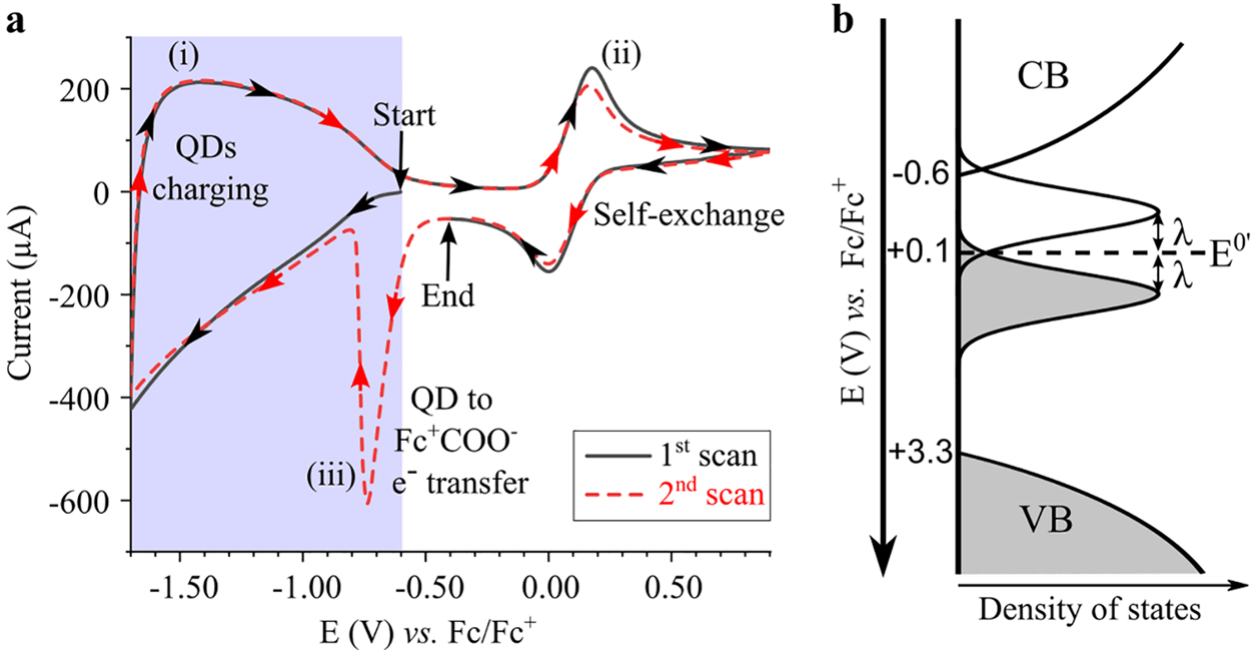
Charge transport pathways in QD/redox ligand assemblies. (a) Cyclic voltammogram (50 mV/s) of the ZnO-FcCOO^–^ film in 0.1 M TBAPF_6_ in acetonitrile. Arrows indicate the direction of the scan. The first scan is the solid black line and the second scan the dashed red line. The start and end points and a shaded area are shown for clarity. The signals have been labeled as (i)–(iii) and are due to (i) QDs charging, (ii) self-exchange, and (iii) QD to Fc^+^COOH electron (e^–^) transfer (see main text). (b) Energy diagram for the ZnO-FcCOO^–^ QDs. The *x*-axis is the density of states, and the *y*-axis is the Fermi level referenced against the Fc/Fc^+^ reduction potential. The energies for the conduction band (CB) edge, FcCOO^–^ ligand formal reduction potential (*E*^0^′), and valence band (VB) edge appear in the *y*-axis and have been obtained from the cyclic voltammogram shown in panel (a); see main text. Empty states are represented in white and filled states in gray. The states of the FcCOO^–^ and Fc^+^COO^–^ are represented as Gaussians for an equal amount of the oxidized and reduced species with a separation between them equal to the double of the reorganization energy (λ).

Figure 2a shows a cyclic voltammogram, a kind of electrochemical spectroscopy, of the QD/redox ligand assembly. This voltammogram contains the fingerprint of the charge transfer processes occurring in the assembly which appear as distinctive signals and have been labeled as (i)–(iii). Here the Fermi level is scanned while the current due to injected/extracted charges is recorded. The measurement starts at −0.6 V (vs Fc/Fc^+^), the initial Fermi level of the film, and the Fermi level is scanned to −1.7 V (vs Fc/Fc^+^), reverted to +0.9 V (vs Fc/Fc^+^), switched back to the initial value (−0.6 V vs Fc/Fc^+^), and the whole scan is repeated again. The black solid line and red dashed line are the first and second scan, respectively. The first part of the first scan (blue shading, black solid line, from −0.6 V to −1.7 V and back to −0.6 V vs Fc/Fc^+^) shows a broad signal, labeled as (i), due to charge injection into the conduction band (CB). Signal (i) is analogous to that obtained in the absence of redox ligand but with the inset shifted ∼0.5 eV to more positive potentials (see Figure S7), which we attribute to the well-known effect that surface dipoles exert on the energy bands of nanocrystals.^19^ Here, the voltage at which the current starts rising determines the position of the CB edge (∼−0.6 V vs Fc/Fc^+^). The second part of the first scan (no shading, black solid line, from −0.6 V to +0.9 V and back to −0.6 V vs Fc/Fc^+^) shows a quasi-reversible peak labeled as (ii) and centered at +0.1 V (vs Fc/Fc^+^). This signal is absent in the ZnO QDs film, appears at a similar position of the FcCOOH (see Figure S8), and points to a clear charge transport pathway across the film inside the bandgap (see next section). The center of this peak determines the FcCOO^–^ ligand formal reduction potential (*E*^0^′) in the film environment.

The second scan (red dashed line) follows accurately the first scan, with the exception of an additional sharp and irreversible peak, labeled as (iii), that starts at the CB edge, is centered at −0.75 V (vs Fc/Fc^+^), and only appears when the film is polarized to potentials above signal (ii). We attribute signal (iii) to electron transfer from the ZnO CB to the ferrocenium carboxylate ligand (Fc^+^COO^–^, the oxidized form of FcCOO^–^) because this transition is thermodynamically favorable. This peak is irreversible and does not appear during the back scan because back electron transfer from FcCOO^–^ to the CB is thermodynamically not allowed. Moreover, signal (iii) is absent during the first scan because initially no Fc^+^COO^–^ is present as all the FcCOO^–^ is in its reduced form, but upon anodic polarization Fc^+^COO^–^ is generated by electrooxidation of FcCOO^–^ as observed in signal (ii). Indeed, integration of peak (ii) shows that the amount of oxidized FcCOO^–^ exceeds the amount of reduced Fc^+^COO^–^, with the difference given by signal (iii).

To support the hypothesis that signal (iii) is due to electron transfer from the CB to Fc^+^COO^–^ we chemically oxidized the FcCOO^–^ redox ligand to Fc^+^COO^–^ by immersion of the QDs-FcCOO^–^ films into a solution containing FeCl_3_. The standard reduction potential of Fe^2+/3+^ (0.22 V vs Fc/Fc^+^)^20^ is higher than that of FcCOO^–^ (0.1 V vs Fc/Fc^+^, Figure S8), meaning the former will oxidize the latter. A cyclic voltammogram of this ZnO-Fc^+^COO^–^ film (Figure S9) shows the presence of signal (iii) during the first scan because Fc^+^COO^–^ is initially present and electron transfer from the CB can occur to the available redox states. Signal (iii), however, does not appear in subsequent scans because all the Fc^+^COO^–^ has been already reduced to FcCOO^–^ during the first scan, and the scan is reversed before the electrochemical oxidation of the redox ligand occurs. This confirms that signal (iii) is due to electron transfer from the CB to Fc^+^COO^–^. We believe this is a general behavior encountered in QD/redox ligand assemblies because similar features have been previously observed in the literature in other QD/redox ligand assemblies but have been left unassigned.^21^ The above analysis by cyclic voltammetry provides a powerful tool to determine charge transfer in QDs that can easily distinguish between electron and hole transfer.

The cyclic voltammogram analysis allows to construct a band diagram of the ZnO-FcCOO^–^ assembly based on the CB edge of the ZnO QDs and the *E*^0^′ of the FcCOO^–^ ligands (Figure 2b). The valence band (VB) edge was determined from knowledge of the bandgap value as determined by steady-state spectroscopy (Figure 1f). In this diagram, the *y*-axis corresponds to the energy level referenced against the Fc/Fc^+^ redox couple and the *x*-axis shows the density of states. The filled states are represented in gray and the empty states in white. The upper and bottom part of the diagram show the presence of the semiconductor energy bands, i.e., CB and VB, respectively. The redox ligand electronic states are centered at *E*^0^′ with its filled and occupied states corresponding to the reduced and oxidized states, respectively. The empty and full bands of the redox ligand are located above and below *E*^0^′, respectively, by a value equal to the reorganization energy.

### Long-Range Charge Transport in QD/Redox Ligand Assemblies

It is interesting to consider the origin of the redox ligand oxidation/reduction resulting in signal (ii) in Figure 2a. This peak exceeds over 2 orders of magnitude the theoretical maximum coverage of a FcCOO^–^ monolayer on a planar surface, and therefore cannot be associated with electron tunneling from the redox ligands in close contact to the ITO substrate. Instead, a long-range charge transport mechanism across the assembly must exist. Transport cannot occur via diffusion of free FcCOO^–^ moieties because they are strongly bound to the ZnO QDs as demonstrated in the first section. Nor it can occur via the CB because it is thermodynamically unfavorable, as the Fermi level lies deep in the bandgap (see Figure 2b). Therefore, it must be a process that occurs from redox ligand to redox ligand, i.e., self-exchange.

**Figure 3:**
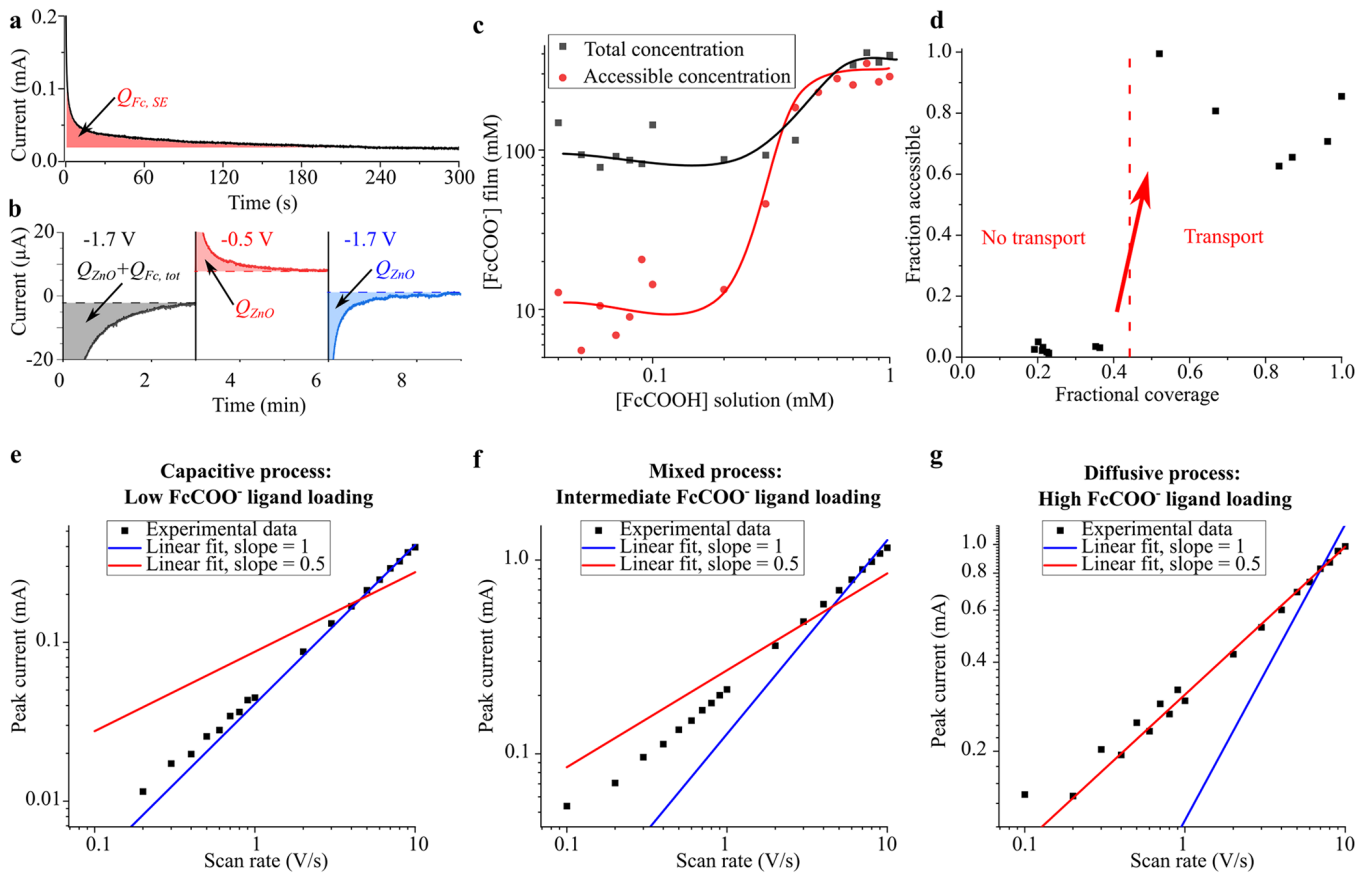
Description of charge transport. (a) Chronoamperogram of the ZnO-FcCOO^–^ assembly used to determine the number of redox ligands participating in self-exchange (see main text). The potential is stepped from −0.5 V to +0.9 V vs Fc/Fc^+^. The integrated area after background subtraction (*Q*_Fc,SE_) is shown in red filling. (b) Triple potential step chronoamperogram of a ZnO-Fc^+^COO^–^ assembly used to determine the total number of redox ligands (see main text). The potential is stepped from equilibrium to the value indicated above each step successively. The integrated area after background subtraction is shown in color filling (first step, gray filling: *Q*_ZnO_ + *Q*_Fc,tot_; second and third step, red and blue filling, respectively: *Q*_ZnO_). (c) Plot of the total (black squares) and accessible through self-exchange (red circles) redox ligand concentration ([FcCOO^–^] film), as measured in (a) and (b) respectively, as a function of the redox ligand concentration in solution ([FcCOOH] solution) during ligand exchange. The lines are guides to the eye. (d) Plot of the fraction of accessible redox ligands as a function of the fractional coverage as determined from (c). The dashed line indicates the percolation threshold. (e–g) Double logarithmic plots of the peak current vs the scan rate obtained from cyclic voltammograms of the self-exchange peak of ZnO-FcCOO^–^ assemblies at three different redox ligand concentrations (shown as the FcCOOH concentration during ligand exchange): (e) 0.1 mM, (f) 0.5 mM, and (g) 1 mM. The symbols are the experimental data, and the blue and red lines are the best fits for a slope of 1 and 0.5, respectively. All measurements were performed in 0.1 M TBAPF_6_ in acetonitrile.

Self-exchange through the redox ligands can only occur if the ligands are sufficiently close to each other on the ZnO QD surface. An interesting question that arises is whether all the redox ligands present in the assembly participate in charge transport and whether we can modulate this transport by changing the concentration of redox ligands. We therefore proceed to quantify both the total number of redox ligands and those that participate in charge transport. This is done by chronoamperometry, an electrochemical technique that measures the number of charges injected into the system when the Fermi level is set to a specified value, Figure 3a,b. The number of redox species involved in this process is then proportional to this charge (Faraday’s law), and the concentration is calculated with knowledge of the film volume (which was determined by profilometry, see Figure S2).

The number of redox ligands involved in charge transport through self-exchange can be obtained by stepping the potential from equilibrium to a value above the self-exchange reaction (signal (ii) of Figure 2a, e.g., +0.9 V vs Fc/Fc^+^) so that all FcCOO^–^ that partake in self-exchange become oxidized. At this potential all current flows via self-exchange and not via the ZnO QDs since the Fermi level is in the bandgap. This chronoamperometry is shown in Figure 3a. The charge given by the integrated current over time (*Q*_Fc,SE_) gives a concentration of 350 mM after correction of background currents (Figure 3a, red filling).

The fraction of redox ligands contributing to charge transport through self-exchange (fraction accessible) can be obtained with knowledge of the total number or redox ligands. This can be achieved by chronoamperometry of the ZnO-Fc^+^COO^–^ assemblies by stepping the voltage to a value inside the CB (−1.7 V vs Fc/Fc^+^, Figure 3b, black curve). The basic idea is that in the CB the whole film becomes conductive and *all* the Fc^+^COO^–^ is reduced. However, this charge also contains the contribution of charge injection into the CB (*Q*_ZnO_ + *Q*_Fc,tot_). To measure this contribution, the potential is stepped outside the CB (−0.5 V vs Fc/Fc^+^, Figure 3b, red curve) and back into the CB; both of these steps give the charge due to charge injection into the CB (*Q*_ZnO_).

The difference in charge between the first and third steps gives then the total number of redox ligands, *Q*_Fc,tot_. The fraction of redox ligands accessible by self-exchange is given by *Q*_Fc,SE_/*Q*_Fc,tot_ and for this particular experiment is 0.9 (i.e., 90% of the FcCOO^–^ ligands in the film contribute to charge transport by self-exchange).

The participation of the redox ligands in charge transport by self-exchange should depend on their concentration in the film. Reducing this concentration yields to an increased distance between the redox ligands lowering their electronic coupling which should affect long-range charge transport. To test this hypothesis, we prepared assemblies with decreasing concentration of redox ligands. The redox ligand concentration was controlled during ligand exchange by adjusting the concentration of FcCOOH in the solution in which the ZnO QD films were immersed. Figure 3c shows the obtained total concentration of redox ligands (black squares) and that involved in charge transport (red circles) as a function of the concentration of FcCOOH in solution during ligand exchange. Figure 3d shows a plot of the fraction of redox ligands that participate in charge transport (accessible fraction) as a function of the fractional coverage. The fractional coverage is calculated by assuming the saturation concentration of Figure 3c (∼350 mM) to be a fractional coverage of 1.0. From Figure 3d it is evident that there exists a concentration threshold at a fractional coverage lying between 35% to 55% above which most redox ligands become available for charge transport. This can be understood in the framework of percolation theory. Percolation theory in 2D cubic lattices predicts a percolation threshold at a fractional coverage of 31%, but this value is increased to 59% for a 3D square lattice.^22^ Our value lies between these two numbers, which is to be expected since the redox ligands are assembled on the surfaces of a 3D assembly of ZnO QDs. Hence the dimensionality of the charge transport can be considered to be intermediate between 3D and 2D.

That long-range charge transport is switched off by decreasing the redox ligand concentration is also seen in the voltammograms. The self-exchange peak (signal (ii), Figure 2a) shows a transition from purely diffusive to purely capacitive behavior with decreasing redox ligand concentration. This is observed from the relationship between the peak current (*I*_*p*_) with the scan rate (ν), Figure 3e–g. *I*_*p*_ scales with ν^0.5^ for the former and with ν for the latter.^20^ At low concentration only the redox ligands close to the ITO substrate are accessible, and charge transport across the film is impeded. These few redox ligands will be charged quickly, as the process does not involve long-range charge transport, and will be complete before the potential is changed to the next potential point during the cyclic voltammetry. In other words, here the charging of the redox ligands is faster than the scan rate, resulting in so-called capacitive behavior, with a current that depends linearly on ν. As the redox ligand concentration increases, charges are allowed to travel across the entire film. This process results in a diffusion layer of oxidized and reduced redox ligands that extends through the film with time. As this process becomes slower than the scan rate, the current will be diffusion limited and scales with ν^0.5^. The peak separation (i.e., separation between the anodic and cathodic peaks) for signal (ii) in Figure 2a progressively increases with the redox ligand concentration (see Figure S10). This is also expected for a transition from a capacitive to a diffusive process.^23^ Assuming infinite electron transfer kinetics to the substrate, the peak separation should be zero for a capacitive process and 57 mV for a diffusive process. The fact that the peak separation values are larger indicate finite electron transfer kinetics.

### Kinetics of the Different Charge Transport Pathways

Charge transport in QD/redox ligand assemblies can occur either via electron hopping through the QDs or by self-exchange involving the redox ligands (see Figure 2a and related discussion). Both of these processes are necessarily accompanied by ion transport to keep electroneutrality. We proceed to independently measure the rate of these three charge transport pathways in order to get a full description of charge transport.

**Figure 4:**
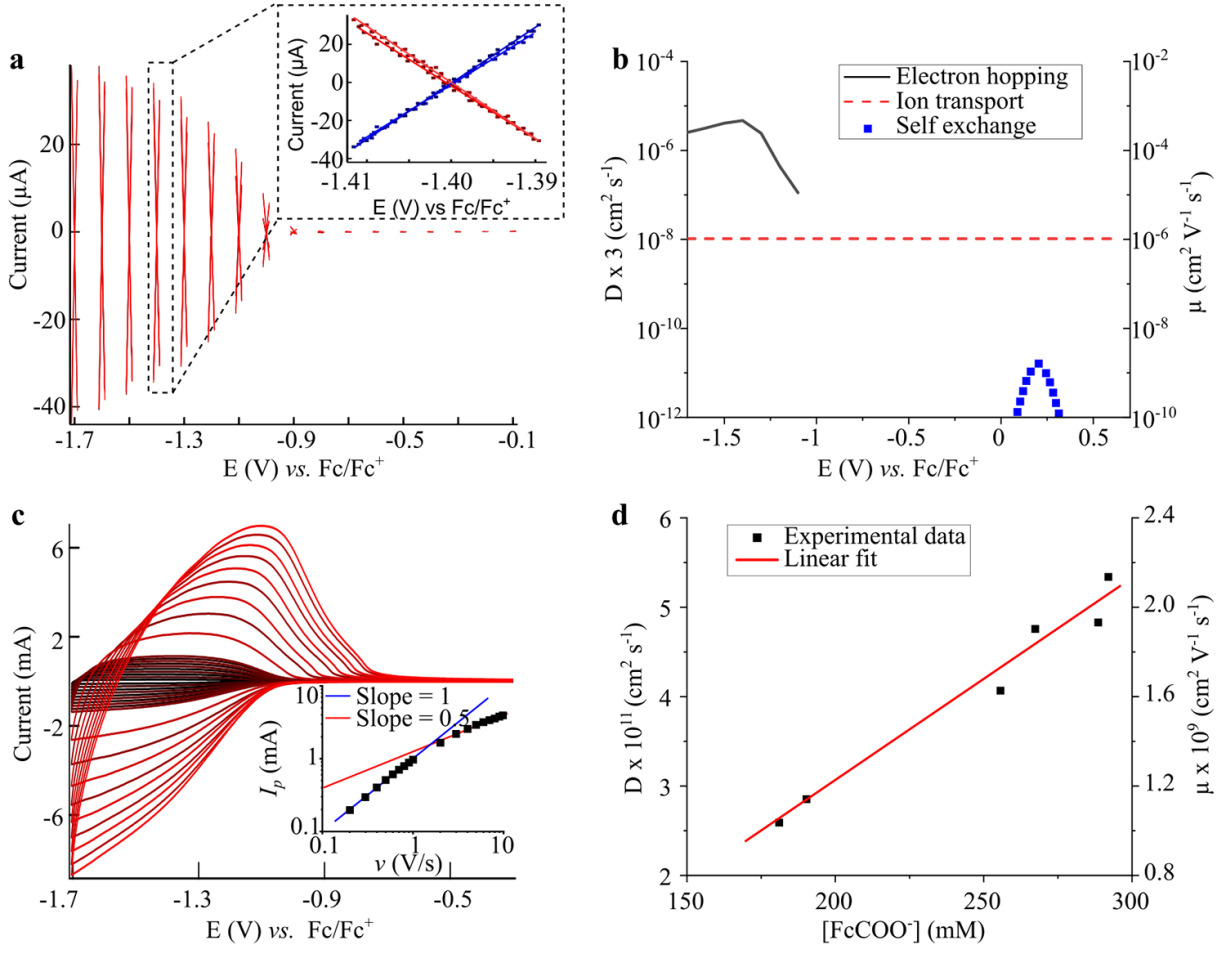
Kinetics of the different charge transport pathways. (a) Electrochemical transistor measurement of the ZnO-FcCOO^–^ assembly. A potential is first applied against the reference electrode and kept until reaching equilibrium (5 s). Then a small voltage sweep (20 mV, see inset) is applied between the source and drain electrodes at a scan rate of 1 mV/s. This process is repeated every 0.1 V from −0.1 V to −1.7 V vs Fc/Fc^+^ and back. The slope of each sweep gives the conductance of the film at the set voltage. (b) Logarithmic plot of the diffusion coefficient (D) and mobility (μ) of electron hopping through QDs (black solid line), ion transport (red dashed line), and self-exchange (blue symbols) as a function of the Fermi level in the ZnO-FcCOO^–^ assembly (see main text for details on how these values are obtained). (c) Cyclic voltammograms at different scan rates (0.1 to 10 V/s) of a ZnO-FcCOO^–^ assembly in the conduction band region. The inset shows a double logarithmic plot of the peak current vs the scan rate (symbols) and best fits with a slope of 1 (blue line) and 0.5 (red line). (d) Plot of the measured diffusion coefficient (D) and mobility (μ) as a function of the redox ligand concentration in the film ([FcCOO^–^]). The red line shows the best linear fit to the data points. All measurements were performed in 0.1 M TBAPF_6_ in acetonitrile.

We determined the rate of electron hopping in the CB by electrochemical transistor measurements using an interdigitated source–drain electrode.^17^ Here, the conductance *G* of the film is measured by applying a small source–drain voltage sweep and measuring the current, via *G* = d*I*/d*V* (Ohm’s law), as shown in the inset of Figure 4a. From the conductance it is possible to calculate the conductivity and, by dividing the conductivity by the integrated charge density, to extract the potential dependent mobility (see Methods for details), which is related to the diffusion coefficient via the Einstein–Smoluchowski relation. To obtain the mobility as a function of the Fermi level this process is performed while applying a bias between the film and the reference electrode and repeated every 100 mV as shown in Figure 4a. The determined mobility as a function of the Fermi level is shown in Figure 4b (black solid line). The mobility increases exponentially as the Fermi level is raised (i.e., the voltage is lowered to more negative values) until it reaches a plateau. At less negative voltages than −0.9 V vs Fc/Fc^+^, the conductivity is below the detection limit.

In a previous work it was shown that the transport of charge compensating electrolyte ions in QD films can be modeled as a diffusive process, in which the diffusion coefficient of the ions is obtained by cyclic voltammetry from the relationship between the peak current and the scan rate of the QD charging signal.^17^ The diffusion coefficient is then calculated from the Randles–Sevcik equation:
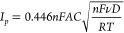
1where *I*_*p*_ is the peak current, *A* the electrode area, *C* the concentration, *v* the scan rate, *D* the diffusion coefficient, and the other parameters have the usual meaning.

Figure 4c shows the voltammograms for charge injection into the ZnO QD CB at different scan rates for a ZnO-FcCOO^–^ film (signal (i) in Figure 2a). A plot of the peak current vs the scan rate (Figure 4c, inset) shows two regions, one at low scan rates (<1 V/s) in which the peak current scales linearly with the scan rate and one at higher scan rates (>1 V/s) in which it scales linearly with its square root. This behavior is due to charging being limited by the capacitance of the film (at low scan rates) or counterion diffusion (at high scan rates), respectively.^17^ This treatment allows to independently determine the rate of ion transport and yields a diffusion coefficient of 7 × 10^–9^ cm^2^ s^–1^ (corresponding to a mobility of 10^–7^ cm^2^ V^–1^ s^–1^), for the tetrabutylammonium cations used in this experiment, in line with the diffusion coefficient determined earlier.^17^ The rate of ion transport is shown in Figure 4b assuming to be independent of the Fermi level (red dashed line).

The rate of self-exchange was obtained in a similar fashion, applying the Randles–Sevcik equation to Figure 3g. This approach gives the electron diffusion coefficient (and mobility) by self-exchange at the redox ligand formal potential (*D*^0^′, 0.1 V vs Fc/Fc^+^), and its dependence on the Fermi level is predicted by eq 2(24) and is shown in Figure 4a (blue squares).
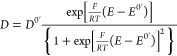
2

We also determined the dependence of the self-exchange rate with the redox ligand concentration. This was only possible above the percolation threshold (∼150 mM), because below this limit self-exchange is governed by a capacitive process, and eq 1 is not valid (see Figure 3e–g). Figure 4d shows that the obtained diffusion coefficients scale approximately linearly with redox ligand concentration. This could be explained by considering self-exchange as a 3D random walk process in which each step occurs over a distance to the nearest neighbor (*r*) with a frequency given by the product of the self-exchange rate constant (*k*_ex_) and the concentration of redox species (*C*):^25−28^
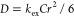
3

The intermolecular distance can be roughly estimated using the law of distribution of the nearest neighbor in a random distribution of particles, in the point-molecule approximation:^29^
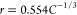
4

This allows to determine the rate constant for self-exchange from the slope of Figure 4d using eq 3, yielding a value of 10^5^ M^–1^ s^–1^, which is 1–2 orders of magnitude lower than reported values for Fc in solution.^30^ There are several factors that could attribute the slightly lower value for the self-exchange rate constant, such the increased intermolecular distance, the lower degrees of freedom of the redox ligands, or the difference in reorganization energy, but investigating this is beyond the scope of the current study.

## Conclusions

We have shown that redox ligands provide a complementary long-range charge transport pathway across QD assemblies. This path can be accessed through the modulation of the Fermi level and redox ligand concentration. The mechanism of transport is through a succession of self-exchange reactions between the redox ligands when they are close enough to have efficient electronic coupling. Long-range charge transport through self-exchange is sharply switched off when the coverage decreases below ∼50%, which was explained by using a percolation model. Above this threshold, the rate of transport increases linearly with concentration and provides an ensemble average of the self-exchange rate constant.

## Methods

### Materials

Zinc acetate (Zn(CH_3_COO)_2_, 99.99% trace metal basis), potassium hydroxide (KOH, 99.99%), tetrabutylammonium hexafluorophosphate (TBAPF_6_, ≥99.0%), ferrocene (Fc, 98%), ferric chloride (FeCl_3_, 97% reagent grade), ethanol (CH_3_CH_2_OH, dry, max. 0.01% H_2_O), methanol (CH_3_OH, ≥99.8% puriss. p.a.), acetonitrile (CH_3_CN, 99.8% anhydrous), and hexane (C_6_H_14_, 95% anhydrous) were purchased from Sigma-Aldrich and used as received. Indium–tin-oxide on glass slides (0.7 mm thick, 7–10 Ohm/Sq) were purchased from MSE Supplies. Ferrocene carboxylic acid (C_11_H_10_FeO_2_, 98%) was purchased from ABCR. Deuterated dichloromethane (CD_2_Cl_2_, 99.8%) and deuterated methanol (CD_3_OD, 99.8%) were purchased from VWR.

### Film Preparation and Ligand Exchange

ZnO QDs were synthesized from a modification of a previously described procedure.^17^ Zinc acetate (0.628 g) was dissolved in ethanol (50 mL) by heating the solution to 60 °C while stirring. When dissolved, a solution of KOH (0.351 g) in methanol (5 mL) was added dropwise (ca. 1 drop per second) and the solution was taken out of the heat. Abundant hexane was added until the solution became turbid. The dispersion was centrifuged for 1 min at 2000 rpm, the hexane removed and the QDs redispersed in 6 mL of ethanol. The QD dispersion was stored at −20 °C and used within one month. QD films were formed by drop-casting the QD dispersion (50 μL) onto indium tin oxide on glass slides (1 × 2.3 cm^2^) and annealed at 60 °C for 1 h. FcCOO^–^ was anchored to the QDs by immersion of the films in a solution of FcCOOH (1 mM, unless stated otherwise) in acetonitrile for 16 h. Unbound FcCOOH was removed by rinsing the films thoroughly with acetonitrile. The ZnO-Fc^+^COO^–^ films were formed by immersing the ZnO-FcCOO^–^ films in a solution containing 0.1 mg/mL of FeCl_3_ in acetonitrile for 1 min and subsequent thorough rinsing with acetonitrile. All procedures were performed inside a glovebox with a water content <0.5 ppm and an oxygen content <0.1 ppm.

### Electrochemical Measurements

Electrochemical measurements were performed with an Autolab PGSTAT128N potentiostat in a three-electrode electrochemical cell setup with a platinum sheet as counter electrode, a silver wire as pseudoreference electrode, and the above-described films as working electrodes. A solution of 0.1 M TBAPF_6_ in acetonitrile was used as electrolyte. The pseudoreference electrode was calibrated throughout the course of the experiments against a ferrocene/ferrocenium (Fc/Fc^+^) couple, giving a constant potential of 0.5 V vs Fc/Fc^+^. All the potentials are reported against the Fc/Fc^+^ couple. All electrochemical measurements were performed under dark conditions and inside a glovebox with a water content <0.5 ppm and an oxygen content <0.1 ppm. All measurements were corrected for cell resistance losses, with a typical value of 50 Ω, as determined by positive feedback.

The rate of electron hopping through QDs was determined by electrochemical transistor measurements. The assembly was deposited on an interdigitated gold electrode with a source–drain gap (*w*) of 50 μm and a total length (*l*) of 21.8 cm. The potential was stepped to a desired value against the reference electrode and, when equilibrium was reached, the voltage was scanned ±10 mV between the source and drain electrodes at 1 mV/s. The slope of the current vs the potential gives the conductance (*G*), which is related to the conductivity (σ) using eq 5:
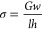
5where *h* is the film thickness.

The mobility can be calculated using eq 6 with knowledge of the charge carrier density (*n*):
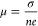
6where *e* is the elementary charge.

The charge carrier density was determined by integration of a cyclic voltammogram.

The mobility is related to the diffusion coefficient through the Einstein–Smoluchowski equation:
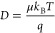
7where *k*_B_ is the Boltzmann constant, *T* the temperature, and *q* the particle (electron) charge.

### Film Characterization

The film porosity was estimated to be 50%, as determined by measurement of the total film volume and knowledge of the volume of ZnO deposited. The film volume was 1.7 × 10^–4^ cm^3^, determined by measuring its thickness (1.5 μm) by profilometry (Dektak 8, Figure S2) and knowledge of the geometrical area in contact with the electrolyte (1.15 cm^2^). The volume of ZnO was 8.5 × 10^–5^ cm^3^, determined from the amount of ZnO deposited and considering a ZnO density of 5.6 g cm^–3^. The amount of ZnO deposited was determined with knowledge of the volume of ZnO dispersion drop casted and the amount of ZnO in the dispersion. The amount of ZnO per volume in the dispersion was determined by drying with a heat-plate a known amount of ZnO dispersion and subsequent weighting. A FEI Quanta 200F scanning electron microscope (SEM) was used to characterize the surface of the QD films on indium–tin-oxide coated glass. To study the distribution of FcCOO^–^ across the film, we measured the Zn and Fe concentration by depth-profile X-ray photoelectron spectroscopy (XPS). We used a Thermo Scientific X-ray photoelectron spectrometer K-Alpha, equipped with a monochromatic Al Kα radiation source and a pass energy of 100 eV for the survey scan, and ion-beam etching unit. The XPS device is equipped with an etching unit which uses Ar+ ions with energy of 1000 eV and a raster size of 2 mm, to remove layers of the QD films. We used the combination of the XPS and etching unit to measure the concentration depth profile of the coated substrates. During the XPS analysis, the spectra of the elements was charge-corrected with the adventitious carbon peak at 284.8 eV. During the XPS measurements, we used the flood gun to compensate for the positive charge.

### Nanoparticle Characterization

The QDs were characterized by transmission electron microscopy (TEM) using a JEOL JEM1400 electron microscope with a field emission gun as the source of electrons operated at 120 keV. Samples were prepared by drop-casting a solution of ZnO-FcCOO^–^ QDs in dichloromethane onto a carbon-coated copper (400-mesh) TEM grid, followed by drying at ambient conditions. Solution NMR spectra were recorded on an Agilent 400-MR DD2 equipped with a 5 mm ONE NMR Probe and operating at 25 °C. ^1^H NMR (399.7 MHz) spectra were collected with a recycle delay of 1 s in deuterated dichloromethane or deuterated methanol. Signals were referenced according to the residual solvent peaks (5.32 ppm for CD_2_Cl_2_ and 3.31 ppm for CD_3_OD).

## Supporting Information Available

Absorption spectrum of ZnO QDs; Spectroelectrochemistry and profilometry of the ZnO QD film; Cyclic voltammetry and steady-state absorption spectroscopy of FcCOOH; Additional electrochemical experiments; XPS of the ZnO-FcCOO^–^ film; Pictures of the ligand exchange workup (PDF)
