# Supplementary material for: Long-Range Charge Transport via Redox Ligands in Quantum Dot Assemblies
Source: ACS Nano. 2022 Dec 14;16(12):21216–24. doi: 10.1021/acsnano.2c09192 (PMC9798906; doi:10.1021/acsnano.2c09192)
Supplement: Supplementary file 1 — nn2c09192_si_001.pdf [file nn2c09192_si_001.pdf]

Supporting Information for

Long-range charge transport via redox ligands in quantum dot assemblies

Yan B. Vogel*, Maarten Stam, Jence T. Mulder and Arjan J. Houtepen*

Optoelectronic Materials Section, Faculty of Applied Sciences, Delft University of Technology, Van der Maasweg 9, 2629 HZ Delft, The Netherlands. Email: y.b.vogel@tudelft.nl; a.j.houtepen@tudelft.nl

Table of Contents:

Pg.

Figure S1. Absorption spectrum of ZnO QDs dispersed in ethanol. --------------- **2**

Figure S2. Measurement of the film thickness by profilometry. -------------------- **2**

Figure S3. XPS of a ZnO-FcCOO$^-$ film. --- **3**

Figure S4. ZnO ligand exchange with ferrocene carboxylic acid. ------------------ **3**

Figure S5. Absorption spectrum of ferrocene carboxylic acid in acetone. -------- **4**

Figure S6. Cyclic voltammetry of the supernatant. ----------------------------------- **4**

Figure S7. Spectroelectrochemistry of a ZnO QD assembly. ------------------------ **5**

Figure S8. Formal potential of ferrocene carboxylic acid. -------------------------- **5**

Figure S9. Cyclic voltammogram of a ZnO-Fc$^+$COO$^-$ film. ------------------------- **6**

Figure S10. Dependence of the peak potentials with the scan rate. ---------------- **6**

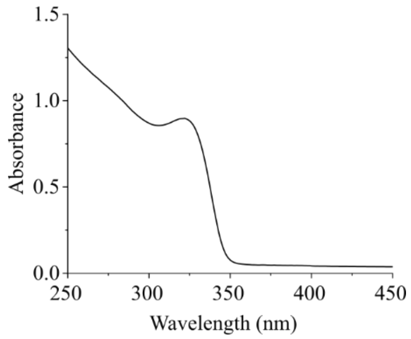

Figure S1. Absorption spectrum of ZnO QDs dispersed in ethanol. *The position of the excitonic peak is used to determine the ZnO QD bandgap (3.86 eV).*

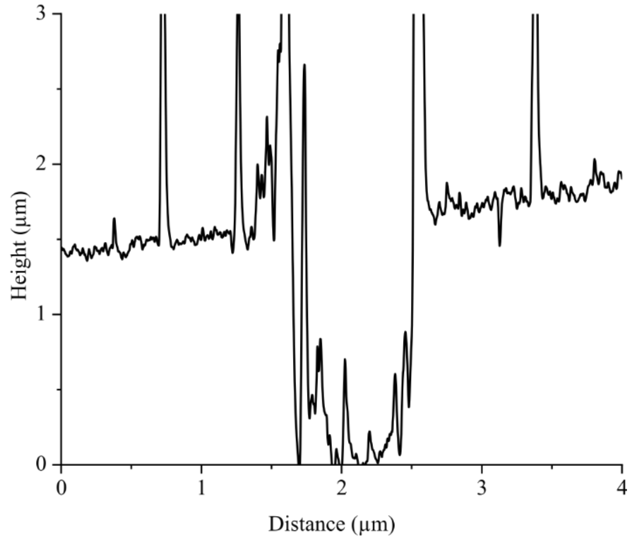

Figure S2. Measurement of the film thickness by profilometry. *Representative profilometry of a ZnO film. The thickness of the film was determined to be 1.5 µm.*

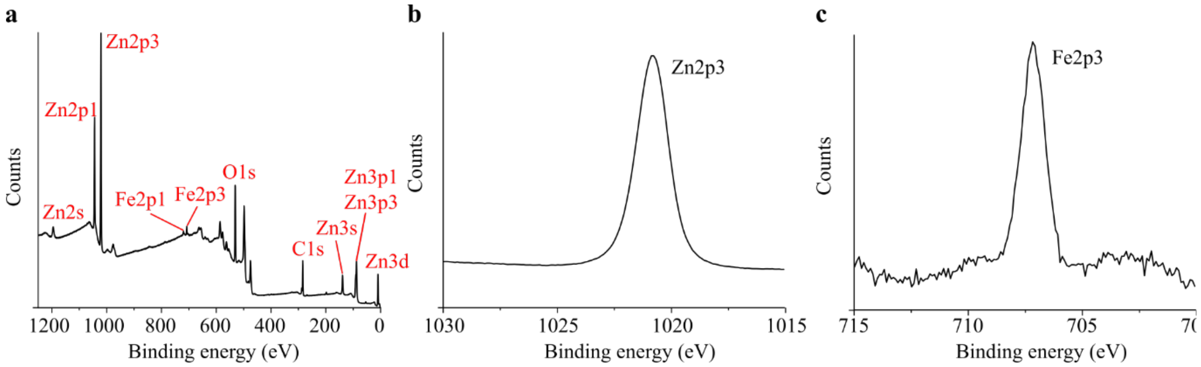

Figure S3. XPS of a ZnO-$FcCOO^-$ film. *(**a**) XPS survey. Representative (**b**) Zn 2p3 and (**c**) Fe 2p3 peaks used to determine the atomic percentage of Zn and Fe, respectively.*

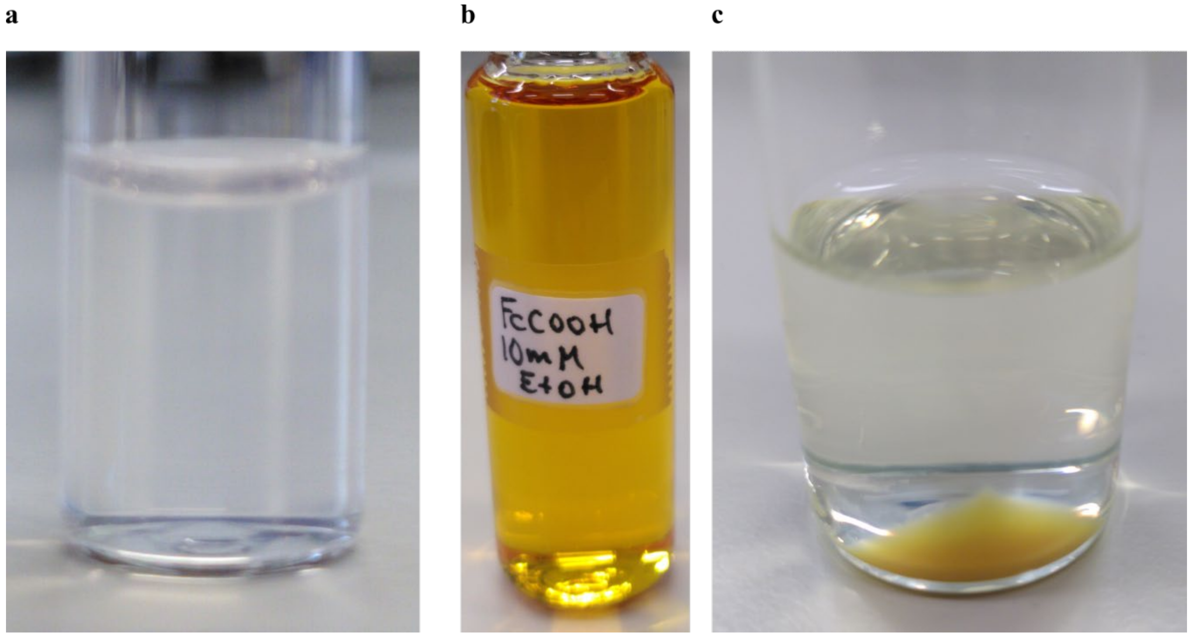

Figure S4. ZnO ligand exchange with ferrocene carboxylic acid. *(**a**) Dispersion of ZnO QDs in ethanol. (**b**) Solution of FcCOOH in ethanol. (**c**) Precipitate of ZnO-$FcCOO^-$ QDs in ethanol after mixing the ZnO and FcCOOH solutions for one hour and centrifugation.*

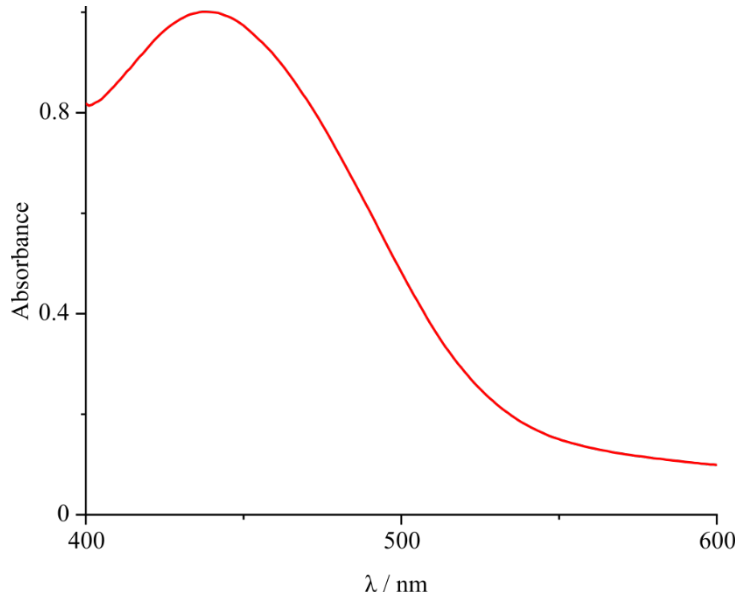

Figure S5. Absorption spectrum of ferrocene carboxylic acid in acetone. *The peak appears at 440 nm.*

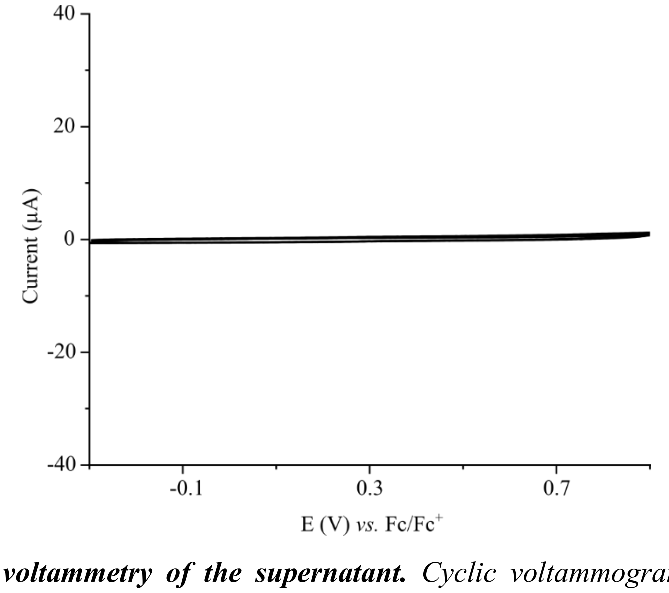

Figure S6. Cyclic voltammetry of the supernatant. *Cyclic voltammogram (20 mV/s) of the electrolytic solution (0.1 M* $TBAPF_6$ *in acetonitrile) used for the electrochemical measurements after being immersed overnight with the ZnO-ferrocene films. No FcCOOH signal is present, proving its absence in solution. A clean ITO was used as the working electrode.*

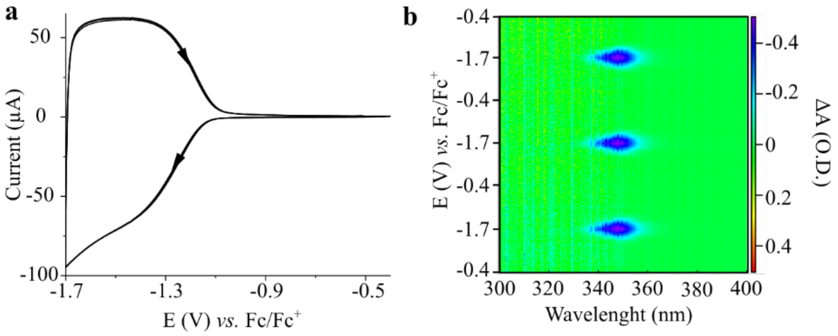

Figure S7. Spectroelectrochemistry of a ZnO assembly. *(**a**) Cyclic voltammetry (20 mV/s, x3 cycles) of a ZnO film in 0.1 M TBAPF$_6$ in acetonitrile. The voltammogram shows reversible and stable charge injection into the conduction band. (**b**) Differential absorption (ΔA) spectra of the ZnO film as a function of the applied potential. The bleach at 350 nm is due to charge injection into the conduction band.*

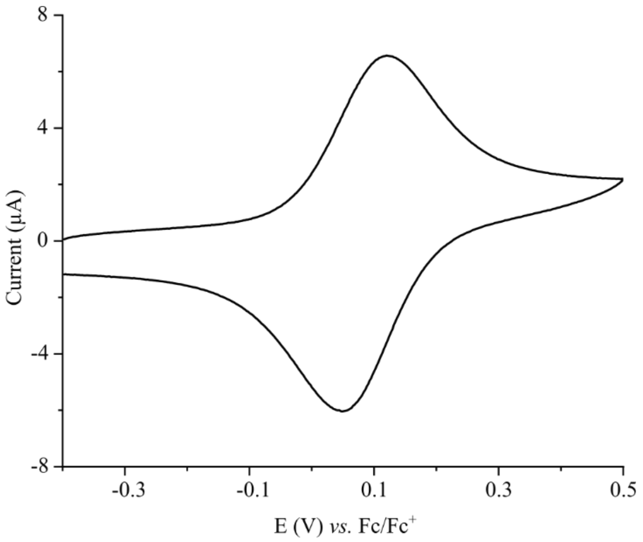

Figure S8. Formal potential of ferrocene carboxylic acid. *Cyclic voltammetry (20 mV/s) of ferrocene carboxylic acid in an electrolytic solution of 0.1 M TBAPF$_6$ in acetonitrile using an ITO working electrode. The determined formal potential is 0.1 V vs Fc/Fc$^+$.*

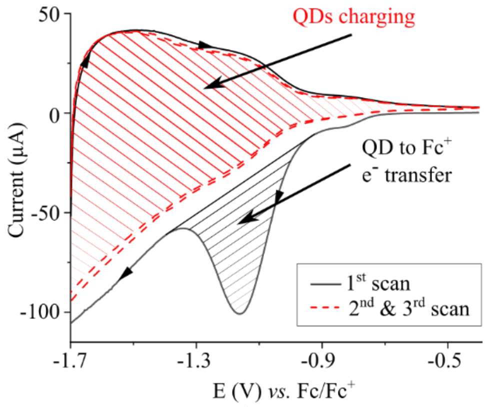

Figure S9. Cyclic voltammogram of the ZnO-Fc^{+}COO^{-} film. *The scan rate is 50 mV/s and the electrolyte TBAPF$_6$ 0.1 M in acetonitrile. The first scan is the solid black line and the second scan the dashed red line. The red and black parallel lines indicate the charge due to QD charging and QD to Fc^{+}COO^{-} electron transfer, respectively.*

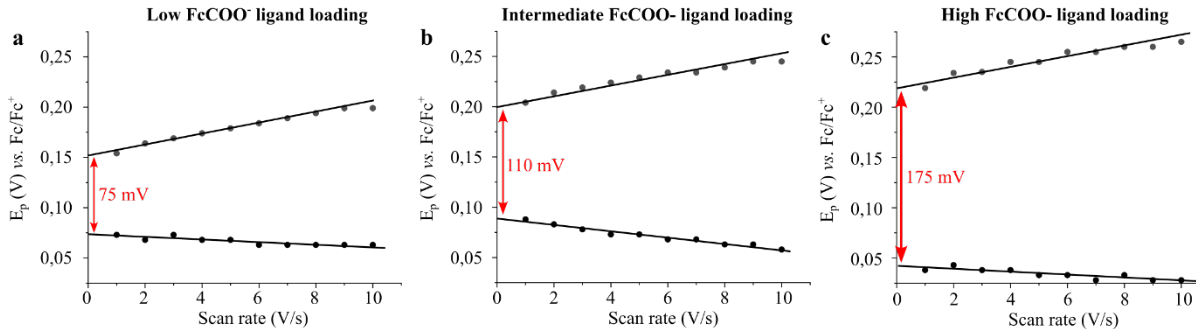

Figure S10. Dependence of the anodic and cathodic peak potentials (E_p) with the scan rate. *The measurements are performed on a ZnO-FcCOO^{-} film at three different redox ligand concentrations (shown as the concentration during ligand exchange): (**a**) 0.1 mM, (**b**) 0.5 mM and (**c**) 1 mM. The redox ligand concentration and peak separation extrapolated at zero scan rate is indicated in each plot.*
